# Hypnotic Modulation of Autonomic Nervous System (ANS) Activity

**DOI:** 10.3390/brainsci14030249

**Published:** 2024-03-04

**Authors:** Giuseppe De Benedittis

**Affiliations:** Department of Neurosurgery, University of Milano, 20125 Milano, Italy; giuseppe.debenedittis@unimi.it

**Keywords:** hypnosis, autonomic nervous system, HRV, EDA, ANI

## Abstract

Hypnosis, a time-honored therapeutic approach, has gained widespread recognition for its effectiveness in addressing a range of psychological and somatic disorders. While its primary effects are mediated by central top–down mechanisms, hypnosis also exerts peripheral influence by modulating the autonomic nervous system (ANS). Psychophysiological measures, such as heart rate (HR) and its variability (HRV), electrodermal activity (EDA), respiratory rate (RR), and the analgesia nociceptive index (ANI), serve as reliable indicators of ANS activity. Studies have consistently demonstrated hypnosis’ ability to significantly impact ANS functions, lowering sympathetic activity and enhancing parasympathetic tone. This effect is particularly pronounced during relaxation procedures and is influenced by mediating factors like hypnotizability and task conditions. Despite methodological limitations, this review highlights the potential of enhanced ANS modulation through hypnosis, including its connections to the central nervous system (CNS), to optimize therapeutic outcomes in patients with psychosomatic disorders associated with ANS dysfunction.

## 1. Introduction

Hypnosis has long been recognized as a valuable therapeutic modality for treating a wide range of psychological and physical conditions, including chronic pain, hypertension, and various anxiety disorders. In addition to top–down mechanisms [1], the effectiveness of hypnosis in addressing conditions that involve altered autonomic nervous system (ANS) function may stem from its ability to effectively reduce psychophysiological arousal and modulate ANS activity [2,3,4].

The autonomic nervous system (ANS) is a crucial component of the peripheral nervous system, regulating involuntary bodily functions like heart rate, digestion, and respiratory rate to maintain homeostasis and respond to environmental changes [5]. It comprises two major branches: the sympathetic and parasympathetic nervous systems, which exert opposing effects on various physiological processes.

While these two branches often function in opposition, their relationship is better characterized as complementary rather than antagonistic. The sympathetic system typically takes the lead in situations requiring quick responses, while the parasympathetic system reigns when immediate action is not essential.

The polyvagal theory (PVT), introduced by Stephen Porges [6], proposes an evolutionary perspective on the autonomic nervous system, particularly the role of the vagus nerve in emotion regulation, social connection, and fear response. It divides the parasympathetic nervous system into two distinct branches: 1. The ventral vagal system (VVS), the most recently evolved part of the parasympathetic nervous system, plays a crucial role in social engagement and affiliative behaviors. It facilitates facial expressions, vocalizations, and eye contact, enabling communication and cooperation with others. 2. The dorsal vagal system (DVS), the more primitive part of the parasympathetic nervous system, governs immobilization behaviors, both “rest-and-digest” and defensive immobilization or “shutdown”. In the face of extreme danger or perceived threat, the DVS can induce a state of tonic immobility (freezing) to avoid predation. While the PVT has gained significant traction, recent studies have challenged its fundamental assumptions. A critical analysis by Grossman [7] concludes that the evidence does not support the role of the “dorsal vagal complex” in freezing as proposed by the PVT, and the dorsal vagal complex “should not be linked to passive defensive behavior”, while the ventral vagal complex, with its role in social engagement and affiliative behaviors, remains a subject of ongoing research.

## 2. Functional Indices of ANS Activity

The most commonly employed psychophysiological measures tap into autonomic bodily effects, encompassing heart rate (HR) and its variability (HRV), electrodermal activity (EDA), and respiratory rate (RR). These indices of autonomic nervous system (ANS) activity align with the relaxation features described by Benson et al. [8] and, either individually or in combination, are often considered neurophysiological hallmarks of the relaxation response [9]. More recently, the analgesia nociception index (ANI), derived from heart rate variability and reflecting relative parasympathetic tone, has emerged as a novel metric in clinical pain management and other clinical settings [10].

### 2.1. Heart Rate Variability (HRV) & Analgesia Nociception Index (ANI)

The rhythmic variation in the interval between successive R–R waves of the electrocardiogram (ECG), reflecting the autonomic nervous system’s (ANS) influence, is termed heart rate variability (HRV). This variation, determined by a balance between sympathetic and parasympathetic activity, reflects the ANS’s current state [9]. In the frequency domain, the HRV waveform is decomposed into its constituent rhythms, and the resulting power spectrum of the tachogram (a series of time differences between consecutive R waves on the ECG) is divided into two primary frequency bands: high frequency (HF) and low frequency (LF). Sympathetic activity is typically associated with the LF band (0.04–0.15 Hz), while parasympathetic activity is associated with the HF band (0.15–0.4 Hz) [11]. The LF/HF ratio serves as an indicator of the relative sympatho-vagal balance [12,13].

Psychophysiologists are increasingly exploring HRV as a crucial marker of physiological and psychological health. For instance, HRV is closely linked to emotional arousal, with high-frequency (HF) activity observed to decrease under acute time pressure, emotional strain, and heightened anxiety states [14,15].

HRV is a sensitive measure that can be affected by artifacts and errors. Respiratory sinus arrhythmia (RSA), a component of HRV, is primarily mediated by parasympathetic influences on the sinus node and is often used as an index of vagal control of the heart. However, there are several caveats to interpreting RSA as a measure of vagal activity. Respiratory factors may play a significant role in determining RSA amplitude. At best, HF variability provides an indirect and imprecise measure of vagal control, and interpretations should be made with caution [16,17].

The analgesia nociception index (ANI), a novel device, provides an objective measure of intra-operative nociception by analyzing heart rate variability (HRV). It is measured on a scale of 0 to 100, where scores above 50 indicate effective analgesia and reflect heightened parasympathetic activity. Conversely, scores below 50 suggest the presence of nociception and an increased dominance of sympathetic activity. Higher ANI scores signify a stronger parasympathetic response within the nervous system [18]. Recent research indicates that ANI has the capability to not only evaluate parasympathetic alterations associated with nociception and analgesia but also to monitor emotional states in conscious patients and healthy individuals during hypnosis. This emerging evidence highlights the potential of ANI as a versatile tool for assessing various physiological and psychological responses [10,18,19,20,21]. While preliminary evidence suggests a potential link between ANI and vagal activity, definitive conclusions are premature. Further research is needed to clarify this relationship and its implications for understanding sympatho-vagal activity, even outside the specific topic of surgical pain.

### 2.2. Electrodermal Activity (EDA)

Electrodermal activity (EDA) is a continuous fluctuation in the skin’s electrical properties, primarily stemming from the changes in eccrine sweat glands. These glands are regulated by the sympathetic nervous system [22], and their activation leads to increased skin conductance, a marker of psychological or physiological arousal. EDA serves as a reliable indicator of both emotional and sympathetic responses [23,24].

External factors such as temperature and humidity affect EDA measurements, which can lead to inconsistent results. In fact, different locations of its measurement can lead to different responses; for example, the responses on the left and right wrists are driven by different regions of the brain, providing multiple sources of arousal; thus, the EDA measured in different places on the body varies not only with different sweat gland density but also with different underlying sources of arousal. These show the complexity of determining the relationship between EDA and sympathetic activity [25].

## 3. Hypnosis Modulation of the Autonomic Nervous System

A substantial body of evidence supports the efficacy of hypnosis in treating various medical conditions associated with autonomic nervous system dysfunction [26,27]. However, the precise impact of hypnosis on the autonomic nervous system (ANS) remains a subject of ongoing debate. Understanding these mechanisms could shed light on the underlying principles of hypnotherapy’s therapeutic effects.

Numerous studies suggest that hypnosis enhances parasympathetic nervous system (PNS) activity [12,28,29,30,31], indicating a relaxation-like effect [32]. Yet, these findings remain inconclusive [33,34,35,36]. Similarly, the effects of hypnosis on sympathetic nervous system (SNS) activity are inconsistent, with some studies reporting decreased SNS activity [12,28,33,35,37,38,39] and others not observing such changes [40,41].

In a recent comprehensive review, Fernandez et al. [42] delved into the vast body of research examining the effects of hypnosis on psychophysiological indices of autonomic nervous system (ANS) activity, particularly those associated with the stress/relaxation response, such as heart rate variability (HRV) and electrodermal activity (EDA). Their analysis specifically examined the influence of hypnotic susceptibility and the impact of specific suggestions or tasks on the observed physiological changes. Their findings, encompassing 49 studies involving 1315 participants, revealed a consistent pattern: hypnosis elicited reductions in sympathetic nervous system activity and/or enhancements in parasympathetic nervous system tone. Notably, the majority of the studies involved healthy volunteers, while only four focused on patients. While the overall findings provide compelling evidence for the ability of hypnosis to modulate ANS activity, the authors highlighted several methodological limitations that warrant attention. These included the use of older studies, employing manual data analyses, the presence of relatively small sample sizes, and the lack of control for multiple comparisons. These limitations underscore the need for more rigorous and standardized research methodologies to further elucidate the mechanisms underlying the hypnotic modulation of ANS activity.

### 3.1. HRV Modulation under Controlled Breath Conditions

To minimize the impact of respiratory suggestions on heart rate variability (HRV) during hypnosis, some studies have utilized neutral hypnosis or hypnosis with controlled respiration. Only a few studies have examined HRV during hypnosis with controlled respiration, and the results have been inconsistent.

In their 1994 study, DeBenedittis and colleagues [12] investigated autonomic changes in ten healthy volunteers, six of whom were highly hypnotizable and four of whom were low hypnotizable. The researchers employed neutral hypnosis conditions (i.e., they avoided direct suggestions of synchronized breathing during hypnosis), which minimized the influence of the vagus nerve on HRV [13]. The findings indicated that hypnosis significantly influenced HRV, shifting the balance of the sympathetic/parasympathetic interaction towards enhanced parasympathetic activity while reducing sympathetic tone. Moreover, a positive correlation was observed between hypnotic susceptibility and autonomic responsiveness during hypnosis, with highly hypnotizable individuals demonstrating a tendency towards a greater increase in vagal efferent activity compared to their less-hypnotizable counterparts.

Contrasting findings were reported by Minonzio et al. [43], who explored autonomic alterations under controlled respiration (respiratory-paced hypnosis) in eleven healthy participants. Autoregressive power spectral analysis of the RR interval and SAP (systolic arterial pressure) variabilities yielded indices of cardiac sympatho-vagal interaction (LF/HF) and vascular sympathetic vasomotor control (LFSAP) (low frequency systolic arterial pressure). No significant changes in the LF/HF ratio emerged between the two hypnosis groups. Hypnosis significantly elevated LFSAP in highly hypnotizable subjects, highlighting the impact of hypnosis on the cardiovascular autonomic profile. This effect was particularly pronounced under controlled respiration, minimizing the influence of respiration rate fluctuations.

### 3.2. Autonomic Changes in Hypnosis vs. Simulation

Limited research has investigated the autonomic responses associated with hypnosis and simulation. Quasi-simulators are often used in hypnosis research to control for the effects of suggestion and compliance. A quasi-simulator in hypnosis is an individual who intentionally mimics the behavior of a hypnotized person without actually being hypnotized. In simulation studies, these individuals are told to simulate the experience of hypnosis by listening to the tape-recorded instructions and were also instructed *not* to allow themselves to become hypnotized.

Bauer and McCanne [44] analyzed heart rate, electrodermal activity, and respiratory rate in six female subjects undergoing hypnosis and six other female subjects feigning hypnosis. Their findings failed to demonstrate substantial differences in physiological responses between the two groups. Intriguingly, both groups exhibited a significant decrease in heart rate during the experimental procedures.

In a subsequent study, Gruzelier et al. [41] delved into the ability of healthy volunteers to modulate auditory tone intensity while under hypnosis and in a simulated state, employing electrodermal activity (EDA) as a measure. Their findings revealed a differential rate of habituation, with slower habituation in the simulation condition and faster habituation in the hypnosis condition compared to the baseline measurement.

## 4. Hypnotizability-Related Modulation of ANS

Despite the methodological inconsistencies in utilizing absolute or normalized LF and HF values, the majority of studies examining the link between hypnotizability and cardiovascular control have focused on the LF and HF components of heart rate variability (HRV) [45]. The findings collectively suggest that hypnotizability levels are associated with distinct patterns of autonomic control. Under prolonged relaxation conditions, both highly and less-hypnotizable individuals exhibit reductions in heart rate (HR). However, highly hypnotizable individuals demonstrate a stronger parasympathetic and a weaker sympathetic contribution to HRV compared to their less-hypnotizable counterparts [45].

By contrast, other studies have reported higher skin conductance (SC) levels in highly hypnotizable individuals compared to moderately and less-hypnotizable individuals, suggesting elevated sympathetic tone [46]. This finding is particularly intriguing considering the absence of significant differences in HR and HRV between the groups. The elevated SC in highly hypnotizable individuals during relaxation could be attributed to their greater attentional engagement in the relaxation task. By contrast, less-hypnotizable individuals may simply disengage from environmental stimuli during relaxation [47].

Moreover, Fernandez et al. [42] explored the relationship between ANS activity and individual hypnotizability levels by comparing ANS measures between high-H and low-H individuals. Their findings indicated that while some studies observed distinct ANS states or reactivity in high-H individuals, the majority found no consistent differences. Specifically, Paoletti et al. [48]; Ray et al. [36], Santarcangelo et al. [49], and Sturgis and Coe [50] found no differences in HR, HRV, and EDA between high-H and low-H individuals. However, O’Connell and Orne [51] reported a correlation between hypnotizability and skin conductance level (SCL) during baseline recordings, with lower SCL observed in high-H individuals compared to low-H individuals. Intriguingly, DeBenedittis et al. [12] found that hypnotizability influenced psychophysiological responses during hypnosis, with high-hypnotizable subjects showing a trend toward an increase of the vagal efferent activity greater than low-hypnotizable subjects.

Electrodermal activity (EDA) and responses have been extensively investigated in relation to hypnotizability, primarily during and after hypnotic induction. While some studies suggest a correlation between hypnotizability and EDA, others have found no consistent differences [47]. A recent study by Kasos et al. [25] examined EDA responses to auditory tones in high- and low-hypnotizable individuals and found that high-hypnotizable individuals exhibited higher left-sided SC responses and faster habituation compared to their low-hypnotizable counterparts. These findings suggest that the relationship between hypnotizability and EDA is complex and may vary depending on the specific context and measures employed.

Overall, the available evidence provides mixed support for distinct autonomic states or reactivity in highly hypnotizable (high-H) individuals, with no consistent differences observed in most cases. These findings suggest that hypnotizability modulates autonomic responses in a complex manner, exhibiting both shared and distinct patterns across different autonomic measures and experimental conditions [52,53].

The inconsistencies between studies are likely attributed to methodological differences. Additionally, some of the observed parasympathetic enhancement could be explained by the relaxation effects induced by hypnosis, as suggested by previous research [27,42]. Further complicating the interpretation is the diversity of autonomic measures used in these studies and the potential for misinterpretation of these measures. To fully understand the mechanisms underlying hypnosis’ influence on the autonomic nervous system (ANS), future research should focus on refining methodologies, standardizing ANS measurement techniques, and clarifying the relationship between hypnosis and ANS activity.

## 5. HRV as a Quantitative Measure of Hypnotic Depth

Diamond et al. [29] examined the correlation between heart rate variability (HRV) and the depth of hypnotic trance. Using a time-frequency analysis of HRV, they compared the continuous self-reported hypnotic depth (SRHD) of 10 volunteers with changes in heart rate, amplitude, and frequency. Their findings revealed significant linear relationships between SRHD and the high-frequency (HF) component of HRV, demonstrating a negative correlation between SRHD and HF frequency and a positive correlation between SRHD and HF amplitude. These results suggest that the responsiveness of the parasympathetic nervous system, as reflected in HRV, could form the basis for a quantitative measure of hypnotic depth. The authors concluded that HRV holds the potential to serve as an effective “hypnometer”, enabling clinicians and researchers to assess hypnotic depth in a dynamic and objective manner.

## 6. Self-Hypnosis and ANS Changes

Self-induced cognitive trance (SICT) has emerged as a novel volitional non-ordinary state of consciousness, drawing inspiration from traditional Mongolian shamanic practices. Oswald et al. [54] unveiled the physiological underpinnings of SICT, revealing a decline in cardiac vagal control, prompting a hyperarousal state of the autonomic nervous system. This physiological response bears striking parallels to the stress response observed in other forms of non-ordinary states of consciousness, such as meditation, and may involve the intricate interplay between cortical and limbic autoregulatory systems.

## 7. Hypnosis Modulation of ANS in Healthy Volunteers

### 7.1. Hypnotic Relaxation

Dunham et al. [31] investigated the effects of hypnotic relaxation on BIS (bispectral) values. The study involved a small trial with three healthy volunteers who were part of a neurofeedback study. In addition to monitoring BIS values, the researchers also conducted autonomic monitoring, which included measuring EDA and HRV. The results of the study revealed a significant reduction in BIS scores, indicating a decrease in cortical arousal, which was accompanied by an increase in parasympathetic neural activation. Furthermore, the study also found a decrease in sympathetic nervous system activity.

### 7.2. Experimental Pain

The cold pressor task triggers an increase in heart rate (HR) along with heightened electrodermal activity (EDA) and systolic blood pressure (SBP). However, subsequent analgesic suggestions effectively reduced HR without influencing the other parameters [55]. In individuals with a high level of hypnotizability (high-H), hypnotic analgesic suggestions also lowered the skin conductance response (SCR) during electrical pain stimuli [56]. This suggests that hypno-analgesia can mitigate the stress response to pain, even though most studies have employed a restricted range of physiological measures.

Terzulli et al. [57] analyzed the effects of virtual reality hypnosis (VRH) on the heat pain threshold among 60 adult healthy volunteers while monitoring several physiological and autonomic functions, During VRH, participants exhibited a clear reduction in their autonomic sympathetic tone, as shown by the lower number of nonspecific skin conductance peak responses and by an increase in the analgesia nociception index (ANI).

### 7.3. Phobias and Induced Emotions

Numerous studies have investigated the responses to suggestions of phobic or aversive stimuli induced through imagery or hypnotic suggestions. The findings revealed a remarkable surge in heart rate (HR) and respiratory frequency (RF) accompanied by a pronounced shift in the sympatho-vagal balance towards a heightened sympathetic tone during hypnotic emotional activation [34,58]. This evidence underscores the ability of hypnosis to evoke autonomic and behavioral responses akin to those triggered by fear-like stimuli, demonstrating its potential for reproducible and controlled manipulation of emotional states.

A study by Kirenskaya et al. [59] found that high-hypnotizable (high-H) individuals exhibit increased heart rate (HR) during emotional recall, while low-H individuals do not experience this effect outside hypnosis. Rainville et al. [60] demonstrated that hypnotically induced emotions can modulate pain perception, with increased HR during induced heat pain when accompanied by negative emotions like sadness and anger compared to hypnotic relaxation alone. These findings suggest that hypnotic suggestions may enhance the intensity of negative emotions, potentially triggering a stress response and hindering pain coping mechanisms.

## 8. Hypnosis Modulation of ANS in Patients

A growing body of research suggests that hypnosis can effectively modulate the autonomic nervous system (ANS), the network of nerves that regulates bodily functions such as heart rate, blood pressure, and breathing. Several studies have compared the ANS responses of individuals undergoing ambulatory interventional procedures with hypnotic manipulations to those receiving medicated sedation. In one study, Baglini et al. [61] examined physiological parameters during coronary angioplasty. They found that the drug sedation group exhibited increases in both low frequency (LF) and the LF/HF ratio, indicating heightened sympathetic activity. By contrast, the hypnosis group displayed no such signs of sympathetic activation.

Another study by Boselli et al. [19] evaluated the analgesia nociception index (ANI), a measure of parasympathetic tone, in patients undergoing axillary brachial plexus blocks for upper limb surgery. Patients in the hypnosis group demonstrated significantly higher ANI scores than those who received standard premedication. This suggests that hypnosis promotes parasympathetic activity, contributing to pain relief.

Chen et al. [62] investigated the short-term physiological impact of a single hypnosis session on patients with major depressive disorder. Heart rate variability (HRV), a measure of ANS balance, was assessed before, during, and after hypnosis. The results indicated that both HF and LF components of HRV increased during and after hypnosis, suggesting an enhanced balance between sympathetic and parasympathetic activity.

Palsson et al. [63] conducted a longitudinal study examining the effects of hypnosis on ANS and clinical symptoms in patients with irritable bowel syndrome (IBS). Patients receiving hypnosis demonstrated significant improvements in symptoms and reduced reactivity to stress, as measured by electrodermal activity (EDA). Notably, heart rate (HR) and blood pressure (BP) remained unchanged.

Excoffier et al. [64] delved into the specific ANS responses to sutures during pediatric emergencies under hypnosis. They observed that the LF/HF ratio decreased during sutures in the hypnosis group compared to the control group. Simultaneously, HF and ANI, indicators of parasympathetic activity, only increased during hypnosis. These findings underscore the potential of hypnosis to effectively regulate ANS function and alleviate pain in pediatric emergencies.

In conclusion, hypnosis emerges as a promising therapeutic modality for modulating the ANS and managing various conditions, including pain and anxiety. Further research is warranted to elucidate the underlying mechanisms and optimize the application of hypnosis in clinical settings.

Table 1 presents a concise overview of the key findings related to the modulation of autonomic nervous system (ANS) activity through hypnosis.

## 9. Discussion, Limitations, and Future Directions

While neuroscience research has primarily been focused on the central mechanisms of hypnosis, recent studies have begun to explore its peripheral effects on the autonomic nervous system (ANS). The autonomic nervous system (ANS) regulates numerous bodily functions, including heart rate, breathing, and blood pressure. Several studies have demonstrated that hypnosis can significantly impact ANS activity, with a consistent reduction in sympathetic activity and an increase in parasympathetic tone. This effect is particularly pronounced during relaxation procedures and can be extended to specific stressors, such as memory recall or phobic stimuli. However, the impact of hypnosis on pain response in healthy volunteers and patients remains less consistent, with some promising findings requiring further replication.

One methodological challenge in studying the effects of hypnosis on the ANS is the evolving nature of heart rate variability (HRV) analysis, a crucial marker of ANS activity. Standardized HRV analysis guidelines only emerged in 1996 [9], potentially affecting the interpretation of earlier studies employing non-standardized methods.

The absence of standardized methodological guidelines across studies hinders the comparability of findings and the identification of consistent mechanisms underlying hypnosis’ influence on the ANS. For instance, the absence of controlled breathing frequency could potentially distort the correlation between high frequency (HF) power and cardiac vagal modulation [12]. Additionally, due to the time-variant and non-stationary nature of the HRV signal, conventional methods such as Fourier transform and AR spectral estimation are inadequate for its analysis. Therefore, wavelet time–frequency analysis emerged as a superior technique, offering enhanced time and frequency resolution while enabling the detection of abrupt amplitude and frequency changes [62]. To further elucidate the mechanisms and clinical applications of hypnosis on the ANS, future research should aim to establish clear and consistent methodological guidelines.

While a substantial portion of research has primarily focused on highly hypnotizable individuals (HVs), limiting the applicability of findings to the general population, future studies should expand their participant pools to encompass a more diverse range of individuals, including individuals with medium hypnotizability (medium H), to comprehensively investigate the effects of hypnosis on ANS function across the entire population spectrum.

The prevalent use of relatively small sample sizes has hindered the statistical power and reliability of the findings, limiting the ability to draw definitive conclusions about hypnosis’ effects on the ANS. Moreover, smaller sample sizes increase the likelihood of type I errors, leading to the risk of false-positive conclusions. To address these limitations, future studies should prioritize larger sample sizes to enhance the statistical power and robustness of evidence, thereby providing a more reliable foundation for understanding hypnosis’ influence on the ANS.

Another notable limitation of the existing literature is the dearth of studies investigating the effects of hypnotic modulation on the ANS in patients and across different clinical conditions, specifically comparing ANS measures before and after hypnotic interventions. Despite its well-established short-term effects on the autonomic nervous system (ANS), the long-term implications of hypnosis on ANS activity remain unexplored. This critical gap in our understanding demands further research to fully comprehend the potential of hypnosis as a therapeutic modality.

To further our understanding of the ANS and its central neural correlates, we must delve deeper into the intricate interplay between these systems. When confronted with real or potential threats in the environment, the body’s autonomic, neuroendocrine, and behavioral responses must operate in concert to ensure adaptation and survival. This complex interplay necessitates input from the limbic forebrain, which serves as a crucial hub for integrating and modulating these responses in accordance with the ever-changing demands of the environment. The ventromedial prefrontal cortex (vmPFC) plays a pivotal role in these regulatory processes. Studies have shown that vmPFC activity is linked to cardiovascular changes during motor tasks, mediated by parasympathetic activity. Furthermore, vmPFC activity plays a significant part in regulating affective and stressful situations [65,66].

Innovative methods have been developed to estimate central autonomic processing, utilizing a novel approach that correlates cardiac-gated fMRI timeseries with continuous-time heart rate variability (HRV). This combined HRV-fMRI approach has demonstrated a high-frequency (HF) correlation with fMRI activity in the hypothalamus, cerebellum, parabrachial nucleus/locus ceruleus, periaqueductal gray, amygdala, hippocampus, thalamus, and dorsomedial/dorsolateral prefrontal, posterior insular, and middle temporal cortices [67].

Expanding upon the groundbreaking research conducted by Fernandez et al. [42], this study offers a comprehensive update and expansion of the field of hypnotic autonomic nervous system (ANS) modulation. By incorporating recent research findings, this study ensures a current and comprehensive understanding of the subject matter, while also integrating the latest evidence.

One significant advancement explored in this study is the investigation of the analgesia nociception index (ANI) as a potential marker of sympathetic/parasympathetic interaction [10,18]. This avenue of research presents a promising new direction for further exploration and understanding.

Moreover, this study emphasizes the critical role of controlled settings, such as breath control and simulator environments, in strengthening the rigor and generalizability of the findings [12,43]. By highlighting the importance of these controlled settings, this study ensures that its conclusions are robust and applicable to real-world scenarios.

Another key contribution of this study is the presentation of the ANS functional index (i.e., HRV) as a potential tool for quantifying hypnotic depth [29]. This innovative approach opens doors for objective assessment in future research, allowing for a more precise understanding of the hypnotic state.

Furthermore, this study delves into the specific mechanisms through which self-hypnosis, particularly in a cognitive trance state, influences ANS activity [54]. By exploring these mechanisms, this study sheds light on the intricate relationship between self-hypnosis and ANS modulation, providing valuable insights into the potential therapeutic applications of this practice.

Lastly, this study ventures into the exciting and promising area of ANS modulation via virtual reality hypnosis [57]. This novel approach represents a cutting-edge development in the field, offering new possibilities for therapeutic interventions and further expanding our understanding of hypnotic ANS modulation.

This study’s conclusion underscores two crucial areas that warrant further investigation in the field of hypnotic ANS modulation. Firstly, there is a need for research to evaluate the long-term effects of ANS modulation through hypnosis on clinical conditions and hypnotherapeutic processes. Understanding the lasting impact of ANS modulation can provide valuable insights into the potential therapeutic benefits and inform clinical practice.

Secondly, this study highlights the importance of gaining a deeper understanding of the dynamic interplay between the central nervous system (CNS) and ANS in response to hypnosis. By unraveling the intricate relationship between these two systems, researchers can uncover the underlying mechanisms and pathways involved in hypnotic ANS modulation. This deeper understanding is crucial for advancing the field and potentially developing more effective interventions and techniques.

Overall, this study builds upon previous research, incorporates recent findings, and offers new avenues for exploration in the field of hypnotic ANS modulation. With its comprehensive approach and innovative contributions, this study is poised to make significant contributions to the field and advance our understanding of this intriguing area of research.

In conclusion, despite valuable contributions from existing research, methodological challenges demand further consideration. Standardizing HRV analysis techniques, establishing clear theoretical guidelines, diversifying participant samples, increasing sample sizes, streamlining experimental designs, and exploring the interplay between CNS and ANS are essential steps towards improving the rigor and generalizability of research in this area [42]. Addressing these methodological limitations will pave the way for a deeper understanding of hypnosis’ mechanisms and clinical implications on ANS function, fostering the development of this promising therapeutic modality.

## Figures and Tables

**Table 1:** The main findings of the hypnotic modulation of ANS activity.

Hypnotic Modulation of ANS			
Authors	Study	PNS	SNS
**Edmonston (1968)** [40]	EDA	≠	≠
**Bauer & Mc Canne (1980)** [44]	HRV, EDA- hypnosis vs. simulation	↑	↓
**Gruzelier & Brow (1985)** [39]	EDA	↑	↓
**Gruzelier et al. (1988)** [41]	EDA-hypnosis vs. simulation	↑	↓
**Griffiths (1989)** [37]	EDA	↑	↓
**De Benedittis et al. (1994)** [12]	HRV-Controlled breathing	↑	↓
**De Pascalis & Perrone (1996)** [33]	HRV	↑	↓
**Gemignani et al. (2000)** [34]	HRV-Induced phobias	↓	↑
**Ray et al. (2000)** [36]	HRV	↑	↓
**Hippel et al. (2001)** [35]	HRV	↑	↓
**De Pascalis et al. (2001)** [56]	HRV	↑	↓
**Palsson et al. (2002)** [63]	HRV-Irritable bowel syndrome	↑	↓
**Baglini et al. (2004)** [61]	HRV-coronary angioplastic	↑	↓
**Diamond (2007)** [29]	HRV	↑	↓
**Aubert et al. (2009)** [28]	HRV	↑	↓
**Santarcangelo et al. (2013)** [55]	HRV	↑	↓
**Chen et al. (2017)** [62]	HRV-depression	↑	↑
**Boselli et al. (2018)** [18,19]	ANI-Healthy volunteers-surgery	↑	↓
**Minonzio et al. (2020)** [43]	HRV-controlled breathing	↑	↓
**Kasos et al. (2020)** [52]	EDA	↑	↓
**Excoffier et al. (2020)** [64]	HRV-pediatric surgery	↑	↓
**Fernandez et al. (2021)** [42]	HRV-EDA-meta-analysis	↑	↓
**Dunham et al. (2021)** [31]	HRV, EDA	↑	↓
**Terzulli et al. (2022)** [57]	HRV-ANI-Virtual Reality Hypnosis	↑	↓
**Oswald et al. (2023)** [54]	HRV-self-induced cognitive trance	↑	↓

PNS, parasympathetic nervous system; SNS, sympathetic nervous system; HRV, heart rate variability; EDA, electrodermal activity; ANI, analgesia nociceptive Index; ↑ increased activity; ↓ decreased activity; ≠ no change.

## Data Availability

Not applicable.
